# Role of path information in visual perception of joint stiffness

**DOI:** 10.1371/journal.pcbi.1010729

**Published:** 2022-11-28

**Authors:** A. Michael West, Meghan E. Huber, Neville Hogan

**Affiliations:** 1 Department of Mechanical Engineering, Massachusetts Institute of Technology, Cambridge, Massachusetts, United States of America; 2 Department of Mechanical and Industrial Engineering, University of Massachusetts, Amherst, Massachusetts, United States of America; 3 Departments of Brain and Cognitive Sciences, Massachusetts Institute of Technology, Cambridge, Massachusetts, United States of America; Johns Hopkins University, UNITED STATES

## Abstract

Humans have an astonishing ability to extract hidden information from the movement of others. In previous work, subjects observed the motion of a simulated stick-figure, two-link planar arm and estimated its stiffness. Fundamentally, stiffness is the relation between force and displacement. Given that subjects were unable to physically interact with the simulated arm, they were forced to make their estimates solely based on observed kinematic information. Remarkably, subjects were able to correctly correlate their stiffness estimates with changes in the simulated stiffness, despite the lack of force information. We hypothesized that subjects were only able to do this because the controller used to produce the simulated arm’s movement, composed of oscillatory motions driving mechanical impedances, resembled the controller humans use to produce their own movement. However, it is still unknown what motion features subjects used to estimate stiffness. Human motion exhibits systematic velocity-curvature patterns, and it has previously been shown that these patterns play an important role in perceiving and interpreting motion. Thus, we hypothesized that manipulating the velocity profile should affect subjects’ ability to estimate stiffness. To test this, we changed the velocity profile of the simulated two-link planar arm while keeping the simulated joint paths the same. Even with manipulated velocity signals, subjects were still able to estimate changes in simulated joint stiffness. However, when subjects were shown the same simulated path with different velocity profiles, they perceived motions that followed a veridical velocity profile to be less stiff than that of a non-veridical profile. These results suggest that path information (displacement) predominates over temporal information (velocity) when humans use visual observation to estimate stiffness.

## Author summary

Stiffness of the arms or legs, the force evoked by displacement, plays an important role in managing physical interaction with objects in the world. Measuring stiffness fundamentally requires physical contact. Nevertheless, previous study showed that humans have a remarkable ability to estimate stiffness solely from visual observation of a computer simulation, with no physical contact. The present study extended that work and found that this ability was robust. In particular, the ability to estimate simulated stiffness was largely unaffected by changing the time course of simulated motion. This was surprising given the extensive prior research reporting that distorting velocity patterns influences motion perception. The results presented in this paper indicate that geometric information (path) predominates over temporal information (velocity) in the perception of stiffness. Given the highly-cited relationship between motor action and perception, it also suggests that the structure of the motor control system we used in the simulations is a reasonable approximation of the neural motor controller. This work provides insight into humans’ representation of motor behavior and how humans interpret and learn from the motor actions of others.

## Introduction

When observing another human, we can only see their overt behavior (e.g., motion); we cannot perceive the underlying neural signals that generate the behavior. Yet humans have an astonishing ability to extract latent information from visually observing the movement of others (for review, see [1]). This impressive ability has been best shown in a plethora of point light animation studies, in which subjects were shown the motion of only a small subset of points on the body. Even from such sparse motion information, humans can easily determine intention from arm movement [2], distinguish emotion from patterns in dancing [3], and identify individuals from gait patterns [4]. In the context of motor learning, Mattar and Gribble [5] showed that subjects who observed the motion of other humans reaching in an unseen force field subsequently performed better when reaching in a similar unseen force field. That study demonstrated humans’ ability to learn about novel force environments solely based on visual observation of kinematics during physical interaction. As a whole, these studies demonstrate that humans can determine latent information from visual observation of motion; extrapolating, it may be possible that humans can interpret underlying control signals used to generate motion too.

Aligned with these results, in previous work we found that humans could infer dynamic properties, specifically joint stiffness, from multi-joint limb motion [6,7]. In the prior study, subjects observed the motion of a simulated stick-figure, two-link planar arm on a computer screen and then estimated its stiffness on a numeric scale. To mimic aspects of human neuromotor control, the arm movement was driven by the superimposition of a hand-space mechanical impedance controller and a joint-space mechanical impedance [8], where mechanical impedance is characterized mathematically by the dynamic relation between motion and a resultant force [9–11]. Results showed that subjects’ stiffness estimates positively correlated with the joint stiffness values used in the control policy, indicating that they could estimate changes in joint stiffness. Remarkably, this was possible without force information or explicit knowledge of the underlying limb controller. It is impossible to unequivocally quantify features such as limb stiffness from motion alone. Thus, humans must have used prior knowledge to estimate latent information from visual observation of motion. To estimate limb stiffness, for instance, their prior knowledge had to be congruent with the relation between limb stiffness and motion produced by the control policy used to drive the simulated limb. However, there are still open questions regarding the form of the prior knowledge used and how it was acquired.

One possibility is that shared resources are used for action execution and action perception [12–14]. The existence of mirror neurons—neurons in the premotor cortex that respond both when a subject performs an action and observes another person perform that same action—supports this notion [15]. Behavioral studies showing that infants [16] and adults [17] learn from observing and imitating the motor behavior of others also indicates a strong link between action and perception. Importantly, this relation appears to be bi-directional [18] that is, both transfer of knowledge from perception to action and from action to perception exists. Considering this relation, studying how humans perceive movement can inform how humans produce movement.

The possibility that humans used knowledge of their own neuromotor system to perform the visual-perception-of-stiffness task suggests that the controller used to simulate the arm motions was an adequate approximation of neuromotor control of upper limb movements. In the previous experiments, the simulated arm movements were produced using a motor controller built on dynamic primitives as proposed by Sternad and Hogan [8,19]. The authors proposed that encoding behavior in terms of primitive actions can simplify the control of the otherwise complex neuromuscular system. Specifically, the prior simulations were a composition of two mechanical impedances, one referenced to a nominal joint configuration, the other referenced to an underlying oscillatory motion. Humans’ ability to estimate stiffness from purely visual information indicates a relation between motor action and motor perception. Moreover, it demonstrates that some of the ‘primitive’ elements believed to underlie motor production (in this case stiffness) also figure prominently in sensory processing and perception.

While the composition of motor behavior with dynamic primitives may serve as a competent model of motor generation and perception, how motor behavior is represented in the nervous system remains an open question. Extensive research shows that parameters such as motion and force are correlated with neural activity, but we have limited understanding of how they are integrated to generate motor commands or interpret motor behavior (for review, see [20]). Furthermore, there is evidence to show that aspects of mechanical impedance are also correlated with neural activity [21]. However, it is unclear how, or even whether, mechanical impedance is encoded in the nervous system. For instance, it is possible that the prior knowledge subjects used to estimate stiffness in our previous work included other aspects of mechanical impedance such as velocity-dependent force (damping). The goal of the study presented here was to further probe the prior knowledge humans use to estimate changes in limb stiffness from visual observation. Specifically, we investigated the role of temporal information in the visual perception of stiffness.

Highly regular patterns exist in the temporal aspects of human motor behavior. When humans generate motion, it is commonly observed that their tangential hand velocity changes logarithmically with the radius of curvature of its path, the so-called 1/3 power law (also commonly referred to as the 2/3 power law, depending on formulation) [22,23]. Furthermore, removing this temporal pattern worsens motion perception. For instance, humans perceive motions that follow the 1/3 power law to be more natural [24] and uniform [25]. They can also anticipate the motion of a system more accurately when it follows the 1/3 power law [26]. Additionally, Dayan et al. [27] found stronger and more extensive neural responses, especially in motor-related areas, when humans perceived motion that followed the 1/3 power-law compared to motion that did not. Maurice et al. [28] also showed that humans can control physical interaction with a robot better when its velocity profile follows the 1/3 power law. Moreover, both velocity magnitude (i.e., speed) and direction are also well-correlated with the motor cortical activity of the brain [29]. Thus, temporal information appears to play a key role in biological motion perception and understanding.

Given the important role of temporal patterns in the generation and perception of human motor behavior, we hypothesized that changing the velocity profile of the simulated arm without changing its path would hinder subjects’ ability to estimate limb stiffness from visual observation. Conversely, if changing the velocity profile had no effect, it would suggest that temporal information is at least subordinate to path information. Here, in Experiment 1, subjects viewed simulated arm motions in which the joint paths varied with simulated stiffness as determined by the dynamic simulation; however, the simulated velocity profiles were modified in three different ways. Counter to our hypothesis, the presence and type of velocity manipulation did not significantly affect subjects’ ability to estimate limb stiffness. Given these results, we conducted a follow-up experiment to determine if temporal information had *any* influence on the perception of stiffness. Thus, in Experiment 2, subjects observed simulated arm motions that all followed the same joint path but with different velocity profiles. Subjects perceived simulations with a veridical velocity profile to be less stiff than that of a non-veridical velocity profile; but there was no difference in subjects’ stiffness estimates of simulations that followed a non-veridical velocity profile. Together, these results suggest that while temporal information may influence humans’ stiffness perception, path information is the predominant factor used by humans to visually estimate changes in limb stiffness. These observations provide further insight into humans’ representation of motor behavior and how humans interpret and learn from the motor actions of others.

## Experiment 1

### Methods

#### Ethics statement

A total of 30 subjects (15 males and 15 females with a mean age of 25.5 ± 5.6 years) took part in Experiment 1 (10 in each of the 3 experimental conditions). Subjects had a variety of educational backgrounds, and none had any prior experience with the experimental task. All subjects gave informed written consent before the experiment. The experimental protocol was reviewed and approved by the Institutional Review Board of the Massachusetts Institute of Technology (MIT IRB Protocol #1508154608). Data of an additional 10 subjects collected as part of a prior study (Experiment 2; [6]), referred to here as the *original* condition, were used for comparison in the statistical analyses of Experiment 1 in the present study.

#### Experimental protocol

**Fig 1:**
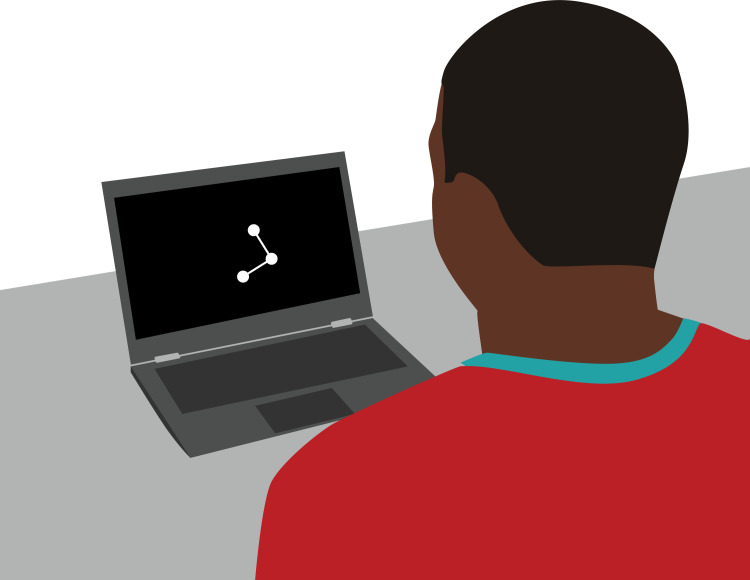
Set up for Experiments 1 and 2. In each trial, subjects were instructed to observe a stick-figure display of a two-link planar arm move along a closed path for 20s and then estimate its stiffness on a numeric scale from 1 (“least stiff”) to 7 (“most stiff”).

In each trial, subjects were instructed to observe a stick-figure display of a two-link planar arm move along a closed path for 20s and then estimate its stiffness on a numeric scale from 1 (“least stiff”) to 7 (“most stiff”) (**Fig 1**). During this time, participants were not restricted from moving their body during the experiment, and they often mimicked the movement using their arm. Note that the endpoint path was not explicitly displayed. After submitting their estimate, subjects self-initiated the next trial. **S1 Video** demonstrates the display that subjects interacted with during these experiments.

Each subject performed 30 trials. Subjects were shown six unique arm motions, each of which was simulated with a different value of elbow stiffness, repeated five times in a blocked manner. The order was randomized within each block. After completing the experiment, subjects were asked to provide a written description of their strategy for estimating “arm stiffness”. The whole experiment lasted approximately 20 minutes.

A custom MATLAB program (The Mathworks, Natick, MA) was used to simulate and display the arm motions and to record subjects’ stiffness estimates. Participants sat approximately 20” in front of a laptop screen (12” w x 7” h) on which the arm motions were displayed. On the screen, the length of each arm link was ~1”.

#### Simulated arm motions

The arm was modelled as a two-link planar manipulator moving in a vertical plane and was driven with a controller comprising two attractors, inspired by the proposal that human motor behavior is composed of dynamic primitives [8,19]. The first attractor was a combination of an oscillatory primitive with mechanical impedance in endpoint coordinates, acting to pull the endpoint along a circular path, and the second was a combination of a fixed-point primitive with mechanical impedance in joint coordinates, acting to pull it to a nominal joint configuration.

**Fig 2:**
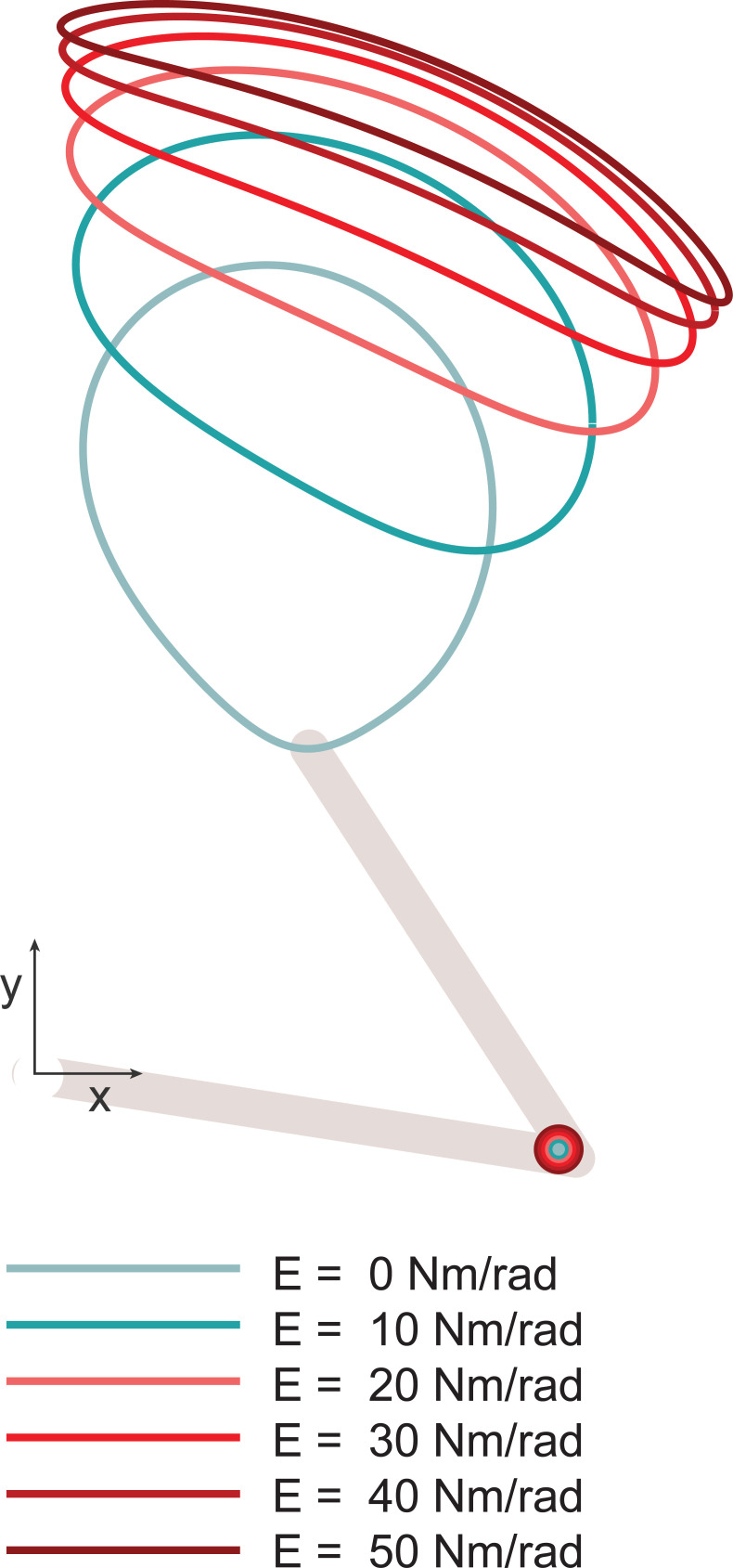
The six endpoint motions of the simulated arm in Experiment 1. Elbow stiffness (*E*) was varied. During the experiments, subjects only saw the moving limb and were not shown the endpoint traces displayed here. The distance between the centroids of the orbital endpoint paths, starting from *E* = 0 *Nm*/*rad* to *E* = 10 *Nm*/*rad*, were 0.24”, 0.15”, 0.09”, 0.05”, 0.03”, respectively, when measured on screen.

The dynamics of this model were described as

$$M(q)\ddot{q}+C(q,\dot{q})\dot{q}+g(q)=\tau$$

where $q,\dot{q},\ddot{q}\in R^{2\times 1}$ are the joint angular positions, velocities, and accelerations, respectively, *M*(*q*)∈*R*^2×2^ is the inertia matrix, $C(q,\dot{q})\in R^{2\times 2}$ are the Coriolis and centrifugal terms, *g*(*q*)∈*R*^2×1^ are the gravitational terms, and *τ*∈*R*^2×1^ are the controller joint torques. The length, mass, center of mass, and moment of inertia parameters for the two links were chosen to match the forearm and upper arm of an average, male human as described in [30]. The controller joint torques *τ* were determined by

$$\tau ={J(q)}^{T}K_{x}(x_{r}-x)-{J(q)}^{T}B_{x}\dot{x}+K_{q}(q_{r}-q)$$

$$x_{r}=\left[\begin{array}{c} .1cos(\frac{20\pi t}{3})\\ .1sin(\frac{20\pi t}{3}) \end{array}\right],q_{r}=\left[\begin{array}{c} \frac{\pi }{4}\\ \frac{\pi }{4} \end{array}\right]$$

$$K_{x}=\left[\begin{array}{cc} 500 & 0\\ 0 & 500 \end{array}\right],B_{x}=\left[\begin{array}{cc} 10 & 0\\ 0 & 10 \end{array}\right],K_{q}=\left[\begin{array}{cc} 0 & 0\\ 0 & E \end{array}\right]$$

where *x*, $\dot{x}\in R^{2\times 1}$ were the endpoint (i.e., hand) positions and velocities, respectively, *J*(*q*)∈*R*^2×2^ was the Jacobian matrix, *x*_*r*_ was the reference endpoint position, which followed a circular path, *q*_*r*_ was the reference joint configuration which was constant, *K*_*x*_ and *B*_*x*_ were the endpoint stiffness and damping matrices, respectively, *K*_*q*_ was the joint stiffness matrix, and *E*∈*R*_≥0_ was the value in the joint stiffness matrix corresponding to the elbow joint. The six unique arm motions were generated by setting *E* = {0,10,20,30,40,50} *Nm*/*rad* (**Fig 2**). The range of elbow stiffness values used was akin to those reported in human studies [31–33]. The dynamic simulation of the arm used in this study was identical to that used in Experiment 2 of our previous study [6].

**Fig 3:**
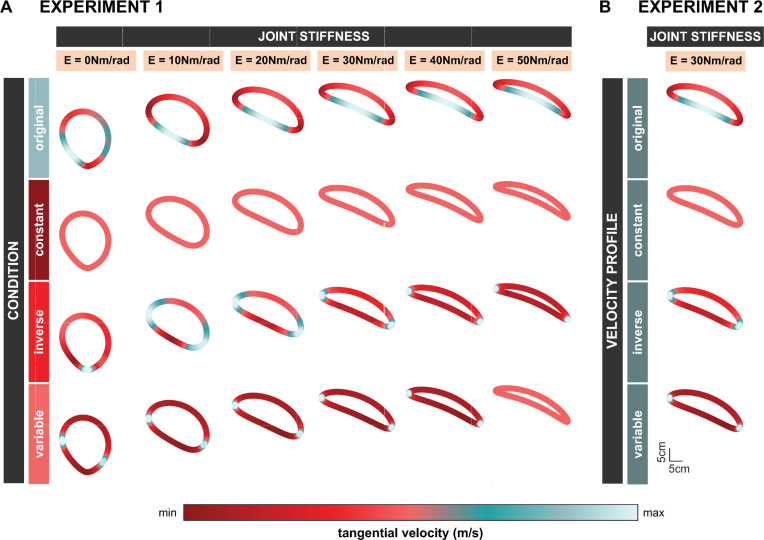
**(A) Simulated arm motions in Experiment 1.** The simulated endpoint velocity profiles for each endpoint path across all conditions in Experiment 1 are shown. In the *original condition*, the velocity profiles followed from the dynamic simulation. In the *constant condition*, the velocity profiles were manipulated to be constant. In the *inverse condition*, the velocity profiles were manipulated to follow the inverse of the veridical power-law relation. In the *variable* condition, the relation between tangential velocity and radius of curvature changed with each simulated stiffness; the velocity profiles did not have a simple velocity-curvature power-law relation (see **Fig 4**). **(B) Simulated arm motions in Experiment 2.** In Experiment 2, the endpoint path (and simulated stiffness) remained the same across the four different arm motions shown to participants, while the temporal pattern along the path differed. The endpoint path was generated with E = 30 Nm/rad; the velocity profiles along the path were chosen from the four conditions in Experiment 1 and are referred to as original, constant, inverse, and variable, accordingly.

**Fig 4:**
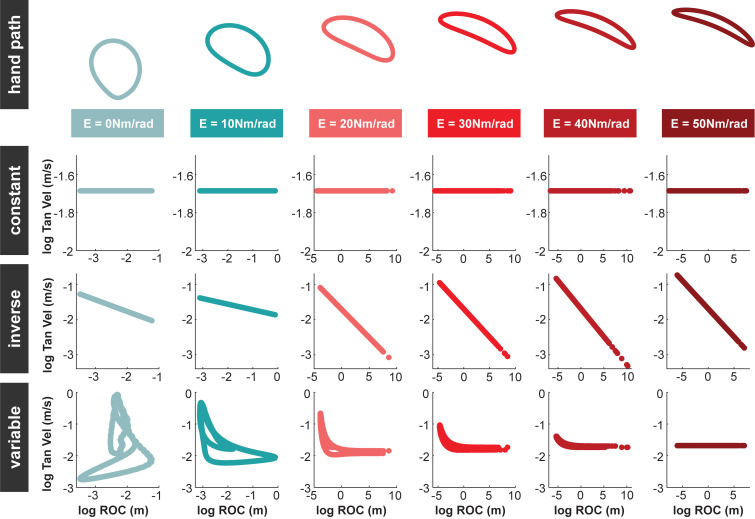
The relation between radius of curvature (ROC) and tangential velocity (Tan Vel) for each endpoint path in the *constant*, *inverse*, and *variable conditions* of Experiment 1. In the *constant condition*, this relation was constant for all endpoint paths. In the *inverse condition*, tangential velocity increased with radius of curvature for all endpoint paths. In the *constant* and *inverse conditions*, the relation was the same for all endpoints path within each condition. In the *variable condition*, this relation differed for each endpoint path. Specifically, the time vector generated to maintain constant tangential velocity for the endpoint path produced from the *E* = 50 *Nm*/*rad* simulation (*constant condition*) was applied to all six different endpoint paths.

While all subjects in Experiment 1 observed the arm move so that it followed the same six endpoint paths (**Fig 2**), the velocity profiles along those paths varied across four experimental conditions (**Figs 3,** S1 **and** S2). Subjects in the *original condition* saw the joints move with a velocity profile governed by the aforementioned dynamic simulation. In the *constant condition*, the tangential velocity of the endpoint, *υ*, was set to a constant value of 0.185 m/s for all six motions (**Fig 4**). In the *inverse condition*, tangential velocity varied as a power of the endpoint path’s radius of curvature *R*(*t*):

$$\upsilon (t)=K{R(t)}^{-1/3}$$

where *K* was the velocity gain tuned to match the period of arm motions shown in the *constant condition* (**Fig 4**). In the *inverse* condition, the speed of the endpoint increased with the curvature of its path. Note that this is the inverse of the typical power law relation between speed and curvature observed in human motor behavior (for review see [34]). The velocity manipulations implemented in the *constant* and *inverse conditions* were chosen for this study because they have been previously proven to affect both motion perception and production [27,28]. In the *variable condition*, the relation between tangential velocity and radius of curvature changed in each condition. The differential equation of the model dynamics was solved using the ODE45 function in MATLAB to obtain a time vector at 1ms resolution. A new time vector was generated to maintain constant tangential velocity for the endpoint path produced from *E* = 50 *Nm*/*rad* (*group 1*). This newly generated time vector was used to generate the arm motion with six different endpoint paths. The position vectors differed for each endpoint path, but the time vector stayed the same. As a result, the relation between speed and curvature differed across endpoint paths and did not follow a power law (**Fig 4**). A video of the different arm simulations subjects observed can be found in **S2 Video**.

While the period of arm motion increased slightly across the six motions displayed in the *constant* and *inverse conditions* (from 3.33 to 3.68 seconds), it was constant in the *original* (3.33s) and *variable* (3.68s) *conditions*.

#### Task instruction

We intentionally did not provide subjects with any details regarding the underlying controller. Prior to the start of the experiment, subjects were not presented with examples of “more” and “less” stiff arm motions. They also did not receive feedback regarding the accuracy of their estimates at any point during the experiment. If a subject was unsure of what the term stiffness meant, they received the following definition: *“Stiffness is the extent to which an object resists deformation or deflection in response to an applied force*. *A stiffer object has higher resistance to deflections than a less stiff object*.*”* In experiment 1, only 8 out of 30 subjects requested and were provided with a definition of stiffness.

#### Self-reported strategy for stiffness estimation

After subjects had viewed all the simulations, they were asked to write down their strategy for estimating stiffness. Subjects were not told that they would have to self-report their strategy before the experiment to prevent potential interference of conscious strategies. Each subject’s response was manually codified with four binary motion features of interest (joint motion, endpoint motion, path information, and temporal information) to quantitatively assess what type of motion information subjects used to estimate stiffness. The criteria used to set the value of each binary feature as either ‘yes’ or ‘no’ are presented in **Table 1**.

**Table 1:** Criteria used to encode the type of information subjects reported using to estimate stiffness in Experiments 1 and 2.

Feature	Value	Criteria
**Path Information**	‘yes’	Use of the following words or phrases: • “distance” • “displacement” • “range of motion” • “angle”
	‘no’	Did not meet criteria for a ‘yes’
**Temporal** **Information**	‘yes’	Use of the following words: • “speed” • “rate” • “acceleration” • “jerk” • “smooth”
	‘no’	Did not meet criteria for a ‘yes’
**Joint Motion**	‘yes’	Use of the following words: • “joint” • “shoulder” • “elbow” • “angle” Hand-drawn picture of the arm with a pointer to at least one of the joints
	‘no’	Did not meet criteria for a ‘yes’
**Endpoint Motion**	‘yes’	Use of the words: • “endpoint” • “hand” Hand-drawn picture of the arm with a pointer to the endpoint
	‘no’	Did not meet criteria for a ‘yes’

#### Statistical analyses

To assess whether the estimates were similar across the four experimental conditions, we conducted a 4 (condition) x 6 (joint stiffness) x 5 (block) analysis of variance (ANOVA) of the arm stiffness estimate. ‘Condition’ was a between-subjects factor, and ‘joint stiffness’ and ‘block’ were within-subject factors. The Greenhouse-Geisser correction was applied to the within-subject factors.

In addition, a linear model of stiffness estimate as a function of joint stiffness was fit to the data for each subject. The coefficient of determination (*R*^2^) was calculated for each subject. The *R*^2^ value represents the fraction of the overall variance of the dependent variable (i.e., stiffness estimate) that can be accounted for by variability of the independent measure (i.e., simulated joint stiffness). It served as a performance measure of each subject’s ability to estimate changes in stiffness. *R*^2^ values closer to 1 indicated better fit of a linear model, and hence, better performance. A one-way ANOVA of the *R*^2^ values with ‘condition’ as a between-subjects factor was conducted. This analysis tested whether the amount of unmodeled variability in subjects’ stiffness estimates differed across experimental conditions. A one-way ANOVA of the fitted slope values with ‘condition’ as a between-subjects factor was also conducted.

Binomial regression was conducted to assess the effect of experiment on the likelihood that subjects reported using path information, temporal information, joint motion, and endpoint motion to estimate stiffness. Linear regression was also conducted to further investigate whether the reported use of the aforementioned motion features could predict subjects’ abilities as quantified by the *R*^2^ values of the stiffness estimate linear fits. Data from the *original condition* was not included in regression analyses since, in that experiment, subjects did not self-report their strategy in writing.

In all statistical tests, the significance level was set to *p* = 0.05. Statistical analyses were performed using SPSS Statistics for Windows, Version 24.0 (IBM Corporation, Armonk, NY).

## Results

### Effects of experimental condition and simulated joint stiffness on stiffness estimate

**Fig 5:**
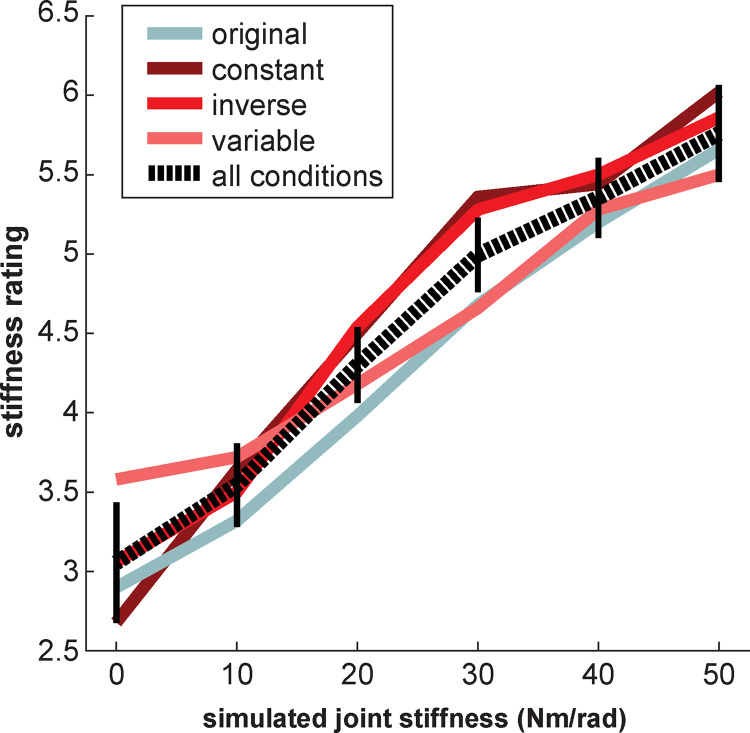
Experiment 1 stiffness estimate results. In all four experimental conditions, there was a significant positive effect of joint stiffness on the arm stiffness estimates. Solid lines show the average arm stiffness estimate across subjects within each condition. The dashed line shows the average stiffness estimate across subjects in all conditions. Error bars represent ± 2 standard errors of the mean.

A three-way ANOVA revealed a significant effect of simulated joint stiffness [*F*(1.64,59.184) = 85.22, *p*<0.001]. Across all experimental conditions, subjects increased their stiffness estimate with the simulated joint stiffness used to generate the arm motion paths (**Fig 5**). However, the remaining effects and interactions were not significant [condition: *F*(3,36) = 0.64, *p* = 0.59; block: *F*(2.67,96.11) = 0.35, *p* = 0.77; simulated joint stiffness x block: *F*(11.40,410.36) = 1.21, *p* = 0.28; simulated joint stiffness x condition: *F*(4.93,59.18) = 1.22, *p* = 0.31; block x condition: *F*(8.01,96.11) = 0.22, *p* = 0.99; simulated joint stiffness x condition x block: *F*(34.20,410.36) = 0.95, *p* = 0.55].

**Fig 6:**
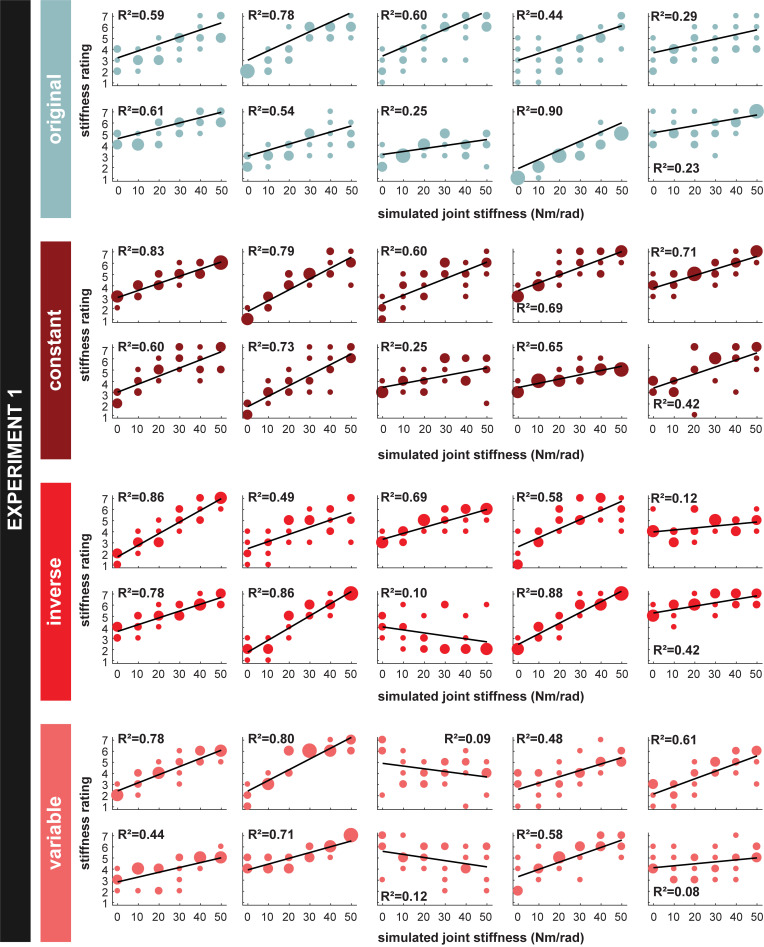
Experiment 1 stiffness estimates for each individual subject. All the individual subjects’ stiffness estimates across simulated joint stiffness in all four conditions. Larger dots indicate greater response frequency. The black lines represent a linear fit of each subject’s data. The coefficient of determination, R^2^, is also reported.

**Fig 7:**
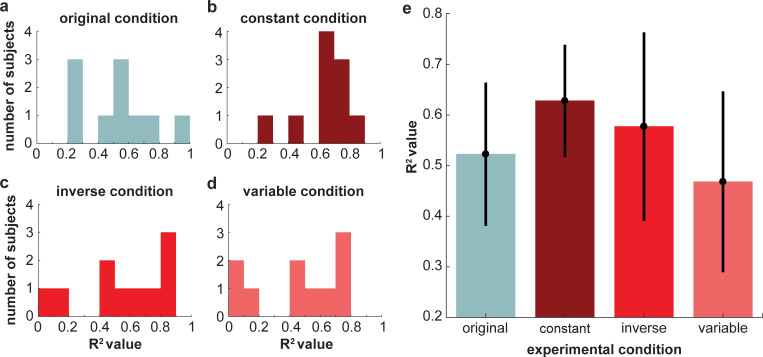
Histograms of the coefficient of determination, *R*^*2*^ in **(a)** the *original condition*, **(b)** the *constant condition*, **(c)** the *inverse condition*, and **(d)** the *variable condition* of Experiment 1. **(e)** Average coefficient of determination, *R*^*2*^, in each experimental condition. Error bars show ± 2 standard errors of the mean. There was no significant effect of experimental condition on the *R*^*2*^ values.

**Fig 8:**
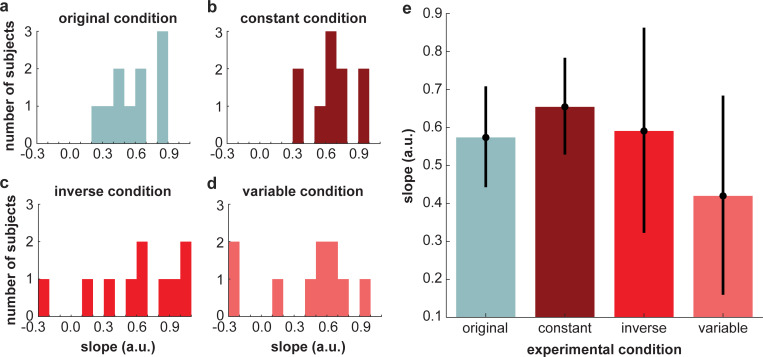
Histograms of the slopes from the fitted linear models in **(a)** the *original condition*, **(b)** the *constant condition*, **(c)** the *inverse condition*, and **(d)** the *variable condition* of Experiment 1. **(e)** Average slope in each experimental condition. Error bars show ± 2 standard errors of the mean. There was no significant effect of experimental condition on the slope values.

**Fig 6** shows each individual subject’s stiffness estimates for every motion path simulated with a different joint stiffness value, along with the linear model fit to each subject’s data and the corresponding *R*^2^ value. The better the linear model fit, the better the subject’s ability to estimate changes in stiffness. Subjects across all experiments varied in their ability to estimate stiffness from motion, as indicated by the overall variation of *R*^2^ values (*M* = 0.55, *SD* = 0.25; **Fig 7A–7D**). A one-way ANOVA revealed that there was no significant effect of condition on the values [*F*(3,36) = 1.04, *p* = 0.39] (**Fig 7E**). A one-way ANOVA of the slopes of the linear fits similarly revealed no significant effect of condition [*F*(3,36) = 0.90, *p* = 0.45] (**Fig 8A–8E**). Across all experimental conditions, the average slope was 0.56 (SD = 0.33). These results indicate that the speed profile of the arm motions had no statistically detectible influence on subjects’ ability to estimate stiffness.

### Effect of experimental condition on motion features used to estimate stiffness

**Fig 9:**
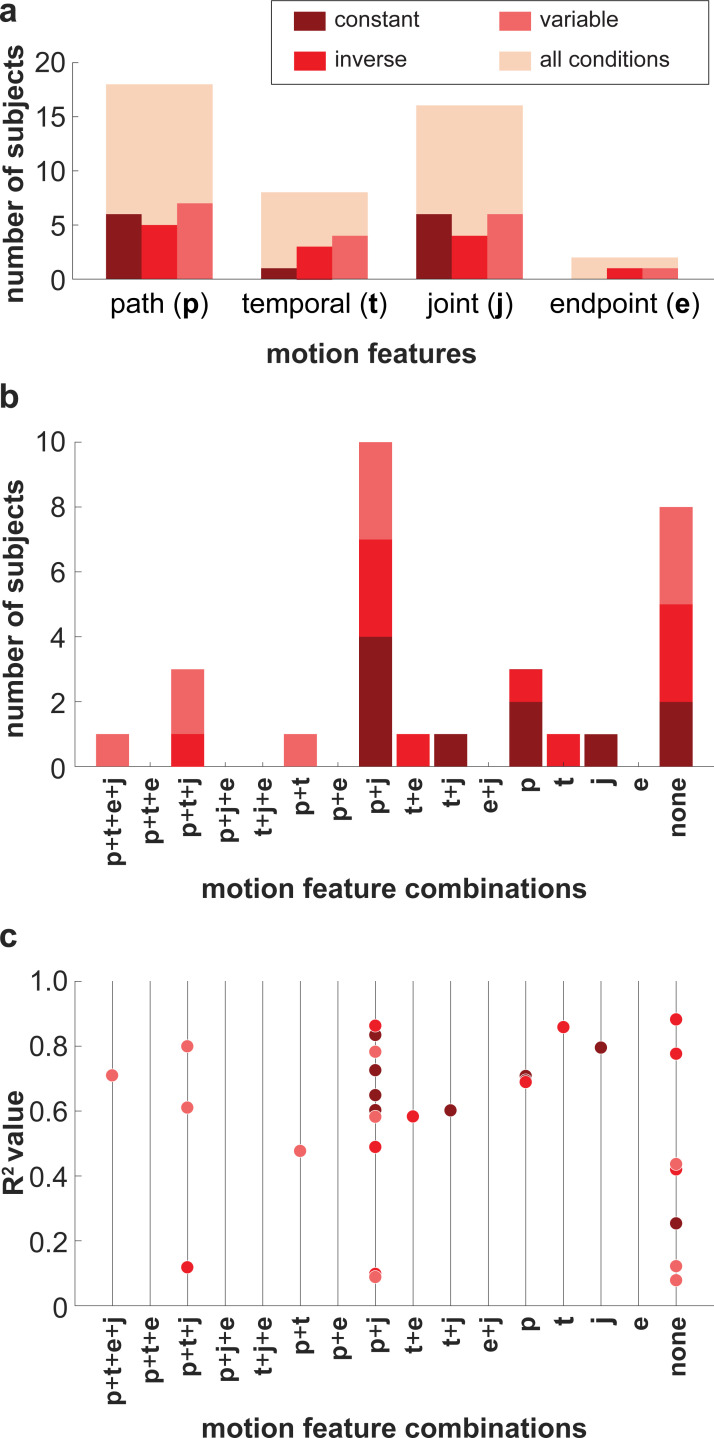
Self-reported strategies in Experiment 1. **(a)** Histogram of motion features that subjects reported using to estimate stiffness. **(b)** Stacked histogram of the sixteen possible motion feature combinations that subjects could have reported using to estimate stiffness (p: path information, t: trajectory information, j: joint motion, e: endpoint motion). A subject who reported using path information (p) and joint motion (j), but not trajectory information (t) and endpoint motion (e), for example, would be counted under the combination ‘p+j’. **(c)** R^2^ values for each motion feature combination that subjects reported using to estimate stiffness. Each circle represents an individual subject. Color differentiates subjects based on the condition they participated in.

To further examine the motion features subjects used to estimate stiffness, analyses of the self-reported strategies were conducted. As seen in **Fig 9A,** more subjects reported using path information (*N* = 18) compared to temporal information (*N* = 8) to estimate stiffness, and more subjects reported using joint motion (*N* = 16) compared to endpoint motion (*N* = 2). Most subjects (*N* = 10) reported using both path information and joint motion (**Fig 9B**). However, a similarly large number of subjects (*N* = 8) did not report using any of the four features.

Results of binomial regression found that experiment did not significantly affect the likelihood that subjects reported using any of the four motion features [path information: *χ*^2^(2) = 0.84, *p* = 0.66; temporal information: *χ*^2^(2) = 2.62, *p* = 0.27; joint motion: *χ*^2^(2) = 1.08, *p* = 0.58; endpoint motion: *χ*^2^(2) = 1.69, *p* = 0.43] (**Fig 9B**). Results of the linear regression also found that none of the four motion features extracted from subjects were significant predictors of a subject’s ability to estimate stiffness (i.e., *R*^2^ value) [*F*(4.29) = 1.90, *p* = 0.94] (**Fig 9C**).

## Experiment 2

The results of Experiment 1 showed that manipulation of temporal patterns did not preclude subjects’ ability to identify changes in simulated joint stiffness. It is still possible, however, that temporal patterns influence the magnitude of humans’ stiffness estimates. Experiment 2 directly tested this possibility.

### Methods

#### Subjects

Ten subjects took part in Experiment 2 (5 males and 5 females with a mean age of 25.6 ± 1.7 years). As in Experiment 1, subjects had a variety of educational backgrounds, and none had any prior experience with the experimental task. All subjects gave informed written consent before the experiment. The experimental protocol was reviewed and approved by the Institutional Review Board of the Massachusetts Institute of Technology.

#### Experimental protocol

The experimental protocol was identical to that of Experiment 1, except that subjects in Experiment 2 were shown four different arm motions, repeated five times in a blocked manner. Each subject performed 20 trials.

#### Simulated arm motions

The endpoint path (and simulated stiffness) remained the same across the four different arm motions, while the temporal pattern along the path differed. As shown in **Fig 3B**, the endpoint path was generated with an elbow stiffness of *E* = 30 *Nm*/*rad*; the velocity profiles along the path were chosen from the four conditions in Experiment 1 and are referred to as *original*, *constant*, *inverse*, and *variable*, accordingly.

#### Task instruction

Task instruction was the same as Experiment 1. In Experiment 2, three subjects requested and were provided with a definition of stiffness.

#### Self-reported strategy for stiffness estimation

As in Experiment 1, after observing all simulations, subjects were asked to write down their strategy for estimating stiffness. Again, subjects were not told that they would have to self-report their strategy prior to the experiment. The same criteria as before (Table 1) were used to codify subjects’ self-reported strategy.

#### Statistical analyses

To assess the effect of velocity profile on stiffness estimate, we conducted a 4 (velocity profile) x 5 (block) ANOVA on the arm stiffness estimate. ‘Velocity profile’ and ‘block’ were within-subject factors. The Greenhouse-Geisser correction was applied to the within-subject factors. Planned comparisons using paired t-tests were made to probe the within-subject effect of ‘velocity profile’.

## Results

### Effects of velocity profile on stiffness estimate with the same endpoint path

**Fig 10:**
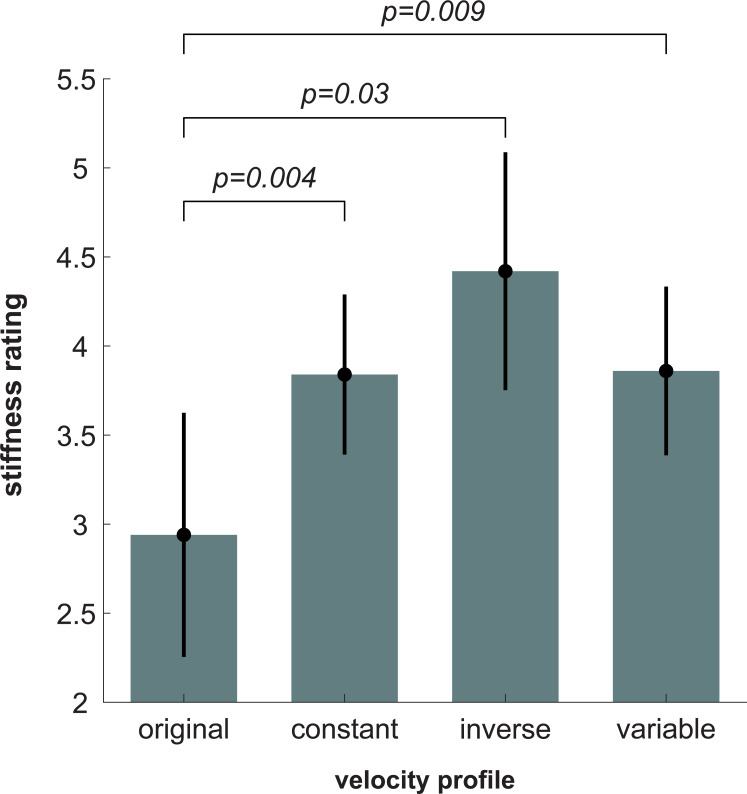
Experiment 2 stiffness estimate results. A significant effect of velocity profile on stiffness estimate was observed. Stiffness estimates for the *original* velocity profiles were statistically smaller than for the manipulated velocity profiles. There was no statistical difference in stiffness estimate between the three manipulated velocity profiles. Bars show the average arm stiffness estimate across subjects for each velocity profile. Error bars represent ± 2 standard errors of the mean.

**Fig 11:**
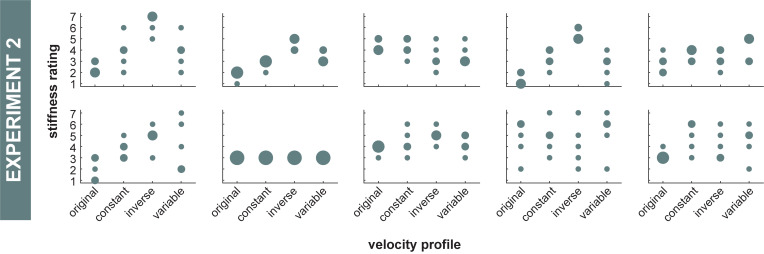
Experiment 2 stiffness estimates for each individual subject. All the individual subjects’ stiffness estimates across the four velocity profiles. Larger dots indicate greater response frequency.

A two-way ANOVA revealed a significant effect of velocity profile on stiffness estimate [*F*(1.37,12.31) = 5, *p* = 0.029 (**Fig 10**). Paired tests revealed that stiffness estimates were significantly lower for *original* velocity profile compared to the three other velocity profiles (*ps*<0.03. There was no statistical difference in stiffness estimate across the other three velocity profiles (*ps*>0.18. The remaining effects and interactions were not significant [block: *F*(2.50,22.53) = 1.08, *p* = 0.37; velocity profile x block: *F*(2.69,24.20) = 0.56, *p* = 0.63]. **Fig 11** shows each individual subject’s stiffness estimates for each velocity profile.

### Self-reported stiffness estimating strategies

**Fig 12:**
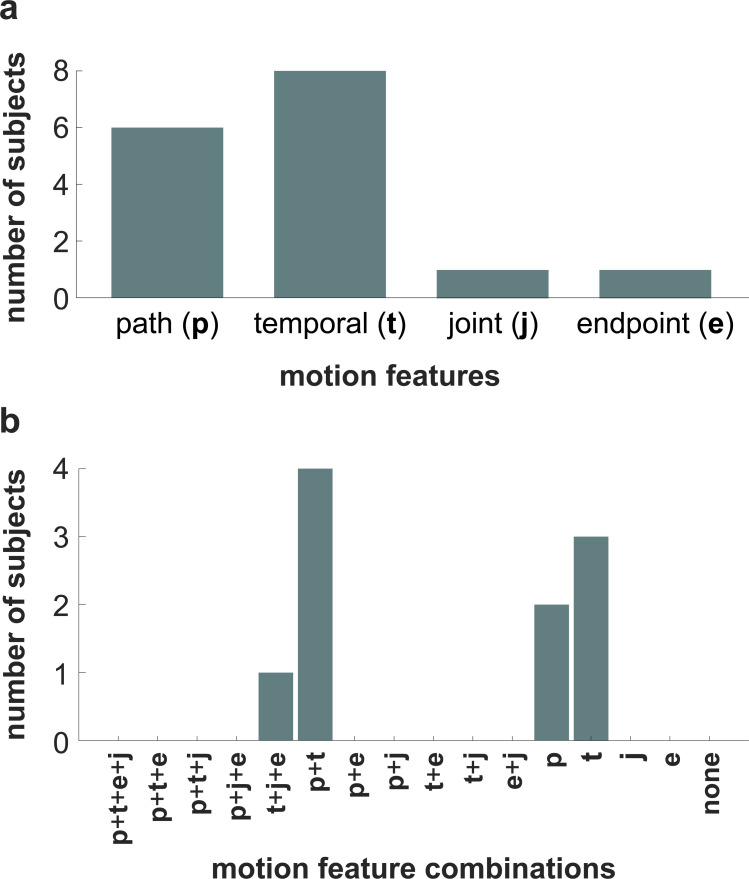
Self-reported strategies in Experiment 2. **(a)** Histogram of motion features that subjects reported using to estimate stiffness. **(b)** Histogram of the sixteen possible motion feature combinations that subjects could have reported using to estimate stiffness (p: path information, t: trajectory information, j: joint motion, e: endpoint motion).

As in Experiment 2, subjects were asked to write down their strategy for estimating stiffness after having viewed all the simulations. Contrary to the results of Experiment 1, in Experiment 2 more subjects reported using temporal information (*N* = 8) as opposed to path information (*N* = 6) to estimate stiffness (**Fig 12A**). However, this was to be expected given that path information did not change across motions in Experiment 2. Moreover, subjects in Experiment 2 rarely reported using either joint motion (*N* = 1) or endpoint motion (*N* = 1) (**Fig 12A**). Rather, many subjects (40%) reported using both path and temporal information (**Fig 12B**).

## Discussion

Given the observed relation between motor action and perception and the dependence of both on velocity, we hypothesized that manipulating the velocity profile would hinder subjects’ ability to estimate stiffness. To test this hypothesis, we simulated arm motion with three new conditions. In each condition, we distorted the velocity profiles of a simulated arm by manipulating the velocity-curvature relation of its endpoint while keeping the paths the same. In all conditions, we found that despite the manipulation of temporal information subjects still increased their arm stiffness estimate with simulated elbow stiffness. Moreover, we did not observe a statistically significant difference in subjects’ ability to estimate limb stiffness across the experimental conditions, including the original condition with veridical velocity profiles. These results emphasize the robustness of our prior finding that humans can estimate changes in limb stiffness from visually observing its motion [6,7]. While the results of Experiment 1 demonstrated that manipulation of temporal patterns did not preclude subjects’ ability to identify changes in simulated joint stiffness, Experiment 2 directly tested the possibility that temporal patterns influence the *magnitude* of humans’ stiffness estimates. Specifically, subjects observed 20 arm simulations that followed the same path (i.e., simulated stiffness), but had 4 different velocity profiles. Analysis demonstrated that subjects estimated simulations with the original velocity profile to be significantly less stiff than simulations with a manipulated velocity profile. Given these results, we conclude that temporal information does influence visual perception of stiffness. However, path information is the predominant factor used by humans to visually estimate changes in limb stiffness. These results provide further insight into how humans interpret and learn from the motor actions of others.

Considerable research has shown that temporal information plays a key role in both the generation and perception of biological motion. And yet, our results show that changing the temporal patterns of the arm motion did not render subjects unable to estimate limb stiffness. However, it is possible that the Likert scale used to quantify subjects’ stiffness estimates was not sensitive enough to capture small differences across experiments. Discrete numeric scales are vulnerable to quantization error, and the variance of quantization error is inversely proportional to the number of estimate options (i.e., the lower the number of estimate options, the higher the noise) [35]. Additionally, the Likert scale is susceptible to response bias, which occurs whenever a person responds systematically on some basis other than what the items were specifically designed to measure [36]. For instance, it is possible that subjects could have different interpretations of the term “stiffness,” leading to increased variability in the strategies used for producing stiffness estimates. If subjects asked what the term “stiffness” meant, they were given the following definition: “Stiffness is the extent to which an object resists deformation or deflection in response to an applied force. A stiffer object has higher resistance to deflections than a less stiff object.” As mentioned earlier, only eleven subjects asked for and were given this definition; However, omitting those subjects from the statistical analysis did not change the significance (or lack of significance) of the results. It is important to emphasize that if our definition of stiffness had been given to all participants, an assessment of whether the instruction potentially biased participants’ performance could not have been made. This motivated our decision to only provide an instruction if explicitly asked. Another common form of response bias is central tendency bias, which occurs when subjects avoid selecting the most extreme results. As seen in **Fig 6**, not all subjects used the full range of the Likert scale, indicating that they were affected by such bias. Some subjects even explicitly reported looking for the most and least stiff simulations; however, those simulations never came. Since central tendency bias effectively reduces the number of estimate options, it further increases measurement noise for a given subject. Nonetheless, we used the Likert scale to quantify subjects’ stiffness estimates to allow comparison of our experimental results with those of our prior studies [6,7]. Furthermore, despite the presence of measurement noise, the results show that the Likert scale measurements were still fine enough to capture the effect of simulated joint stiffness on stiffness estimate in Experiment 1. Moreover, it allowed us to determine the effect of velocity profile on the magnitude of subjects’ stiffness estimates in Experiment 2. We conclude that, if the experimental procedure did affect subjects’ stiffness estimating ability, the effect was minimal.

**Fig 13:**
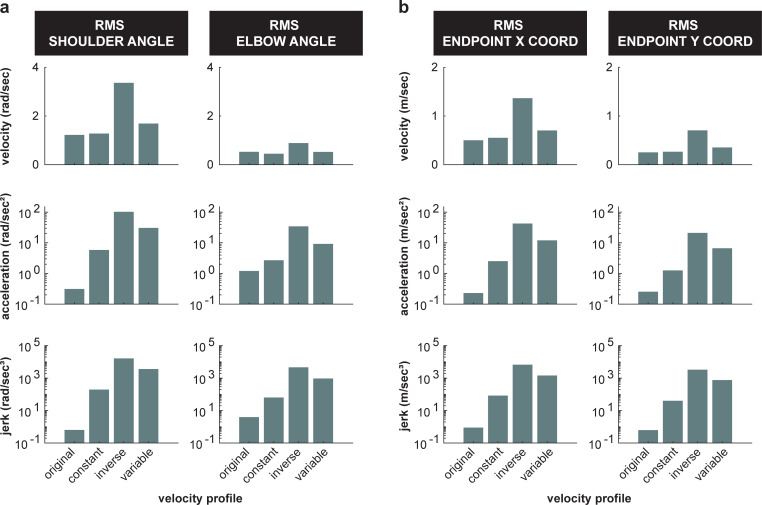
RMS of temporal motion features in Experiment 2. All 4 velocity profiles’ RMS of velocity (rad/s), acceleration (rad/s^2^), and jerk (rad/s^3^) for 1 cycle of motion in **(a)** joint coordinates, and **(b)** endpoint coordinates.

In Experiment 2, where subjects observed simulations of the same endpoint path produced from the same simulated joint stiffness, subjects estimated the stiffness of the veridical velocity profile to be less than that of the other three non-veridical velocity profiles. If subjects solely used path information to estimate stiffness, we would expect subjects to have chosen the exact same stiffness estimate. While one subject did exhibit this behavior (**Fig 11**), that was not the trend for all subjects (**Fig 10**). Upon comparing **Figs 10** and **13**, it is seen that subjects’ stiffness estimates may be related to the acceleration or jerk of the simulated motion. Thus, participants possibly used one of these temporal metrics to estimate changes in stiffness, as the path-based metrics typically used were not available. Consistent with these results, it has been shown that minimizing mean-squared jerk yields the widely-observed 1/3 power law relation between speed and curvature [22,23,37,38], and moreover motions that follow the 1/3 (veridical) power law are perceived to be more natural [24] and uniform [25]. Here, subjects perceived these more ‘natural’ and ‘uniform’ movements to be less stiff. Throughout the literature (for review see [34]), it has often been shown that velocity encodes information about biological vs non-biological motion. Given that minimizing jerk leads to a velocity profile commonly observed in biological motion, subjects may have adopted this heuristic when estimating stiffness (**Fig 13**). As a result, subjects rated biological velocity profiles to be less stiff than non-biological velocity profiles (**Fig 10**). While participants had an overall cognitive bias to estimate smoother (i.e., less jerky), veridical motions as less stiff, this bias was likely not learned within the time course of the experiment. There was no statistically significant effect of trial nor a statistically significant interaction between trial and velocity profile on the stiffness ratings in Experiment 2. Ultimately, while this temporal information may have an effect on subjects’ ability to estimate stiffness, it is still subordinate to path information.

**Fig 14:**
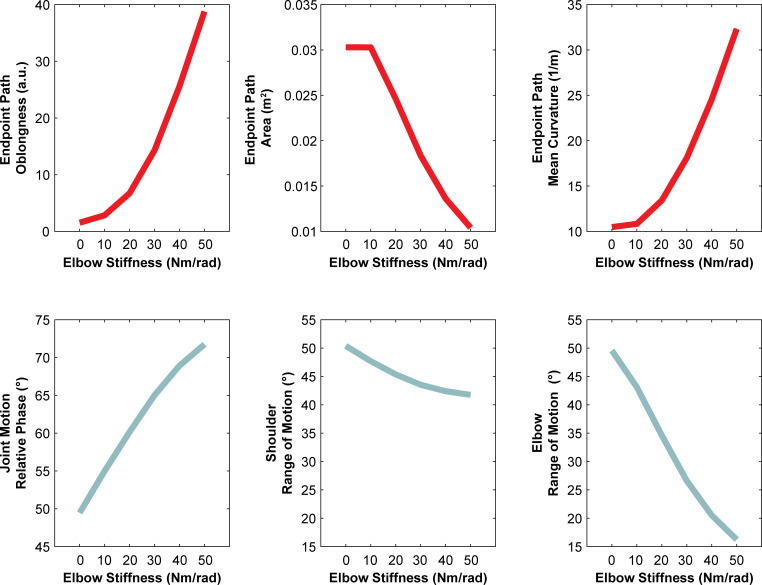
**Change in motion related features with simulated elbow stiffness** (adapted from [6]) **(A)** Endpoint path oblongness was defined as the ratio of the eigenvalues of the covariance matrix of the endpoint path in Cartesian coordinates. **(B)** Endpoint path area was defined as the area enclosed by the end-point path in Cartesian coordinates. **(C)** Endpoint path mean curvature was defined as the average inverse of the radius of curvature (see Methods). **(D)** Joint motion relative phase was defined as the relative phase between the elbow and shoulder motion and was calculated using cross-correlation analysis. **(E)** Shoulder range of motion (ROM). **(F)** Elbow range of motion (ROM).

Given that stiffness is the relationship between force and motion and no information related to force was provided, participants must have used some set of path-based heuristics to estimate changes in stiffness. It is important to emphasize, however, that we cannot claim what path-based heuristic(s) participants used as there are a number of possibilities (e.g., endpoint path oblongness, endpoint path area, endpoint path mean curvature, joint motion relative phase, shoulder range of motion, or elbow range of motion) (**Fig 14**). Inherently, our findings are consistent with the idea of attribute substitution [39]. Attribute substitution occurs when people unconsciously make a judgement about a target attribute based on simpler and/or more accessible substitute attributes [39]. The process of attribute substitution reflects the assumptions or cognitive biases that an individual holds about the relation between the target and substitute attribute(s). In our study, participants used some motion attribute(s) (substitute attributes) to estimate changes in stiffness (target attribute). Here, Experiment 1 (**Fig 5**) demonstrates that overall participants held a “correct” bias or assumption about how changes in motion related to changes in joint stiffness; moreover, this bias was relatively consistent across individuals (**Fig 6**). Importantly, this assumption of the relation between motion and stiffness was unaffected by manipulations of temporal information. However, Experiment 2 shows that participants still held a bias or assumption about how temporal attributes related to stiffness when differences in path were not present. Thus, we conclude with the novel finding that path-related attributes predominate over time-related attributes when humans estimate stiffness. Future work will investigate what specific path-related attributes are used.

Subjects’ self-reported estimating strategies further support the finding that the use of path information predominated over temporal information when subjects visually estimated changes in limb stiffness. To estimate arm stiffness, in Experiment 1, more subjects reported using motion features that contained path information than those that contained temporal information. Moreover, velocity profile did not significantly influence whether subjects reported using path or temporal information (**Fig 9A**). It is possible that subjects only chose to ignore temporal information because it was not veridical. On the other hand, in Experiment 2, subjects were forced to use temporal information as the simulated endpoint paths were indistinguishable. Thus, contrary to Experiment 1 where few subjects (~23%) reported using temporal information (**Fig 9A**), in Experiment 2 many subjects (80%) reported using temporal information (**Fig 12A**). Interestingly, of the 30 subjects in Experiment 1, only 1 (~3%) reported using the words “jerk” or “smooth”; however, 4 of the 10 subjects (40%) in Experiment 2, reported using the words “jerk” or “smooth”. These results, together with **Figs 10** and **13**, suggest it is likely that subjects did use temporal information to estimate stiffness when differences in path information were removed. Even so, the fact that velocity profile manipulations did not hinder subjects’ ability to estimate stiffness in Experiment 1 indicates that the influence of temporal information is at least subordinate to path information.

Many subjects reported strategies that did not clearly include any of the four motion features (**Fig 9B**). Examples of such strategies reported interest in “flexibility of the object,” “rigidity of the motion,” and “how constrained the object’s motion appeared”. To avoid codifying subjects’ open-ended, written responses, we could have asked subjects to choose from a pre-determined list of motion features. While this may have simplified the coding of their estimating strategies, it would have also increased the chances of response bias (e.g., choosing features from the list for the sake of it). Moreover, Table 1 is not an exhaustive list of the motion heuristics subjects could have used. **Fig 14** highlights other path-based motion heuristics that were related to the joint stiffnesses of the observed simulations. This study did not aim to determine which path-based heuristics subjects used to estimate stiffness. Rather, we examined subjects’ use of path and temporal information; the features chosen in Table 1 helped us to do so. Furthermore, it is also important to be mindful that no matter how subjects reported their estimating strategy, subjects could have reported features that they did not actually use (or omitted reporting features they did use). An inability to clearly articulate what strategy they used could also signal that estimating stiffness using solely observation of motion is an implicit process rather than a conscious one. This could explain why the combination of reported motion features did not affect the stiffness estimating performance (**Fig 9C**).

Nonetheless, the fact that subjects could successfully estimate changes in limb stiffness is remarkable, and even more so is the fact that this ability is robust to manipulations of temporal information (**Experiment 1**). The same limb motion can be generated with an infinite number of stiffness values, making it fundamentally impossible to unambiguously estimate stiffness from motion alone. This begs the question, how are humans able to estimate limb stiffness from just the visual observation of motion? The results from individual subjects indicated that they did not achieve task success by simply guessing how changes in motion related to changes in stiffness. If they did, we would expect the relation between their stiffness estimates and the simulated joint stiffness values to be significantly negative for some subjects. However, this was not the case. While 3 out of 40 subjects, in Experiment 1, did have a negative slope in the linear model fit to their estimating data, the corresponding R^2^ values were very low (<0.12) (**Fig 6**). Excluding these subjects from the statistical analyses did not change the significance or non-significance of any results. Moreover, calculation of the Pearson’s correlation coefficient for each of these three subjects confirmed that there was no significant, linear correlation between stiffness estimates and simulated joint stiffness (*p*>0.05). If these subjects did guess, it was a fruitless strategy. Instead, subjects must have drawn on prior knowledge to successfully perform the experimental task. Furthermore, that prior knowledge had a similar relation between limb stiffness and motion as the one resulting from the control policy used to drive the simulated limb.

Extensive evidence from behavioral and neuroimaging studies shows a strong coupling between action generation and perception processes in the human nervous system [18,40–42]. Thus, one possible explanation is that subjects may have used implicit knowledge of their own neuromotor control system to successfully estimate the changing stiffness of the simulated arm. In fact, a study of adaptation to a force field during reaching by a proprioceptively deafferented patient found that an internal representation of limb dynamics could be updated by visual information alone [43]. As mentioned previously, the controller used to drive the simulated arm intentionally emulated aspects of human neuromotor control, based on the proposal that motor actions are built from dynamic primitives using the compositionality of mechanical impedance [8,19]. As described in this theoretical framework, composing motor actions from motion primitives alone is insufficient. Inclusion of mechanical impedance, minimally defined as stiffness, is also required to control interaction or even the prospect of interaction. Studies of kinematically constrained movements corroborate this notion [44,45]. For instance, an oscillatory motion primitive combined with a mechanical impedance is a competent model of human interaction control during crank-turning (i.e., moving with a circular kinematic constraint) [45] and during human-robot physical interaction [46]. This model offers a plausible explanation of how humans manage physical interaction with their high degree of skeletal redundancy [8,19]. Outside the domain of human motor control, this computational model has proven to be an effective control strategy for kinematically redundant robots [47]. The fact that subjects could estimate stiffness from observing motion alone further supports the role of impedance in the generation of motor actions. Moreover, the fact that subjects could do this without explicit knowledge of the underlying controller suggests that the internal representation used by humans to interpret the motion of others is consistent with the form of the computational model proposed to describe the generation of motion. However, further research is needed to determine the degree of congruency between subjects’ motion generation and perception needed to perform this experimental task. Nevertheless, the importance of the agreement between the simulated controller and subjects’ motor perception should not be undervalued.

Whatever internal model or process was used to perform the task, regardless of its exact form, it must have incorporated stiffness. The novel contribution of the study presented here is that the representation of stiffness in this model is based primarily on path information. At first blush, this appears to contradict the extensive evidence indicating the important role of temporal patterns in biological motion perception. One feasible explanation for this apparent discrepancy is that here, subjects were asked to judge a mechanical property of a simulated arm, not anticipate or predict its kinematic behavior. Smoothness is a measure of predictability, and prior work has shown that maximizing smoothness (i.e., minimizing mean-squared jerk) yields a widely-observed power law relation between speed and curvature [22,23,37,38]. Hence, manipulating the temporal pattern of an object’s motion to eliminate the power law relation between speed and curvature decreases its predictability. While this would likely affect a human’s ability to track or anticipate the object, explaining the results of studies such as Kandel et al. [26] and Maurice et al. [28], it would not necessarily impair their judgement of mechanical properties, especially if such estimations are made based on the geometric features of movement.

Another plausible reason why distorting temporal patterns did not inhibit subjects’ ability to estimate joint stiffness was the presence of multiple moving bodies. Here, subjects were presented with motion of a two-link arm, and more subjects reported using joint motion (*N* = 16) rather than endpoint motion (*N* = 2). In prior studies where modulation of the speed-curvature power law relation reportedly affected motion perception, subjects were only shown a single moving body, or part of its trajectory, which is analogous to displaying only endpoint motion [25–27]. It is possible that spatial structure may be prioritized over temporal structure when multiple bodies are moving. For instance, using the point light animation paradigm with a walking figure, Hirai and Hiraki [48] found that manipulation of spatial structure (i.e., randomizing the start point of a particular point light, while keeping its velocity vector the same) had a substantially greater effect on the brain’s response in the occipitotemporal regions, than manipulation of the temporal structure (i.e., scrambling the frames in the point light animation).

Ultimately, the fact that our results suggest that path information (i.e., geometry) is the predominant motion characteristic in humans’ internal representation of stiffness is consistent with the foundation of mechanics. Only with the postulates of geometry derived by Euclid could the likes of Newton, Lagrange, and Hamilton describe the motion of objects and deduce their own postulates regarding the forces that cause it (for review see [49]). Geometry is fundamental to mechanics. Therefore, it is reasonable that internal models used to interpret dynamics during action perception are predominantly based on geometry.

## Conclusion

To conclude, here we showed that humans can correctly infer changes in limb stiffness from nontrivial changes in multi-joint limb motion. This result was robust despite manipulations of the arm’s endpoint velocity profile. However, when path information was indistinguishable, veridical velocity profiles were perceived as less stiff than non-veridical velocity profiles. While other researchers have shown that manipulation of the velocity profile may hinder humans’ ability to anticipate kinematics, we show that it does not hinder humans’ ability to estimate stiffness. These observations suggest that stiffness, or more generally mechanical impedance, of the limb is encoded in an internal model used to perform this task. Moreover, these results provide new insight into how humans interpret the motor actions of others and suggest that path, not trajectory, information is more important to humans when estimating stiffness from motion. This exploration of how humans extract latent features of neuromotor control from kinematics provides new insight into how humans interpret the motor actions and interactions of others.

## Supporting information

S1 Video Experiment’s graphical user interface. A video showing an example of the graphical user interface used to allow subjects to initiate a trial, observe a simulation, and rate its stiffness.
(MP4)

S2 Video Simulated arm motions. A video showing the 24 different arm simulations that subjects saw in Experiments 1 and 2.
(MP4)

S1 Fig Simulated arm tangential velocities. The simulated endpoint tangential velocities of all 24 experimental paradigms (4 velocity conditions x 6 stiffness conditions) are shown. Each plot shows a single cycle.
(EPS)

S2 Fig **(A) Simulated arm velocity magnitudes.** The simulated endpoint velocity magnitudes of all 24 experimental paradigms (4 velocity conditions x 6 stiffness conditions) are shown. Each plot shows a single cycle.
(EPS)
